# Holistic tool for ecosystem services and disservices assessment in the urban forests of the Real Bosco di Capodimonte, Naples

**DOI:** 10.1038/s41598-022-20992-0

**Published:** 2022-09-30

**Authors:** Antonello Prigioniero, Bruno Paura, Daniela Zuzolo, Maria Tartaglia, Alessia Postiglione, Pierpaolo Scarano, Sylvain Bellenger, Anna Capuano, Eva Serpe, Rosaria Sciarrillo, Carmine Guarino

**Affiliations:** 1 Department of Sciences and Technologies, University of Sannio, Via De Sanctis snc, 82100 Benevento, Italy; 2 Department of Agriculture Environment and Food, University of Molise, Via De Sanctis snc, 86100 Campobasso, Italy; 3 Ministry of Culture, Museo E Real Bosco Di Capodimonte, Via Miano, 80131 Naples, Italy

**Keywords:** Ecosystem ecology, Ecosystem services, Urban ecology

## Abstract

A tool for urban forest Ecosystem services (ES) and disservices (ED) assessment has been developed to visualize (i) overall ES and ED value, (ii) ES–ED trade-off and (iii) explore principal influences in ES and ED provision. The Real Bosco di Capodimonte (RBC) (Naples, Southern Italy) has been chosen as a case study. ES and ED linked to urban forest plant cover were: biodiversity, carbon storage, gross and net carbon sequestration, lessen runoff, oxygen production, air pollution removal, UV effects reduction, pollen-related allergenicity risk, and volatile organic compounds (VOCs) emissions. A phytosociological survey was conducted and biodiversity value was evaluated. ES and ED were assessed by i-Tree Eco model and Index of Urban Green Zones Allergenicity (I_UGZA_). Results showed that 441 different plant species occur in the RBC and the most represented genera are *Quercus* and *Trifolium*, while the largest family was Asteraceae. Carbon storage and pollution removal were highest in natural forest, while remaining ES were greater in managed forest areas. Highest value for VOCs emission and allergenicity were assigned to managed and natural forest, respectively. Managed forest scored the highest ES–ED value, while managed grassland scored the lowest. Results highlighted the greater influence of plant cover structure in overall ES and ED provision levels, and management influence considering the same type of plant cover. The model could be a valuable tool for ES and ED effective management generally applicable in urban forests.

## Introduction

Excessive urbanization and the accompanying climate change of our time are associated with the emergence of increasingly aggressive environmental problems^1–4^. It is well known that vegetation in urban (public and private) areas such as parks, streets, urban forests and gardens, has the capacity to mitigate these problems and provide a range of benefits conceptualized as provisioning, regulating, cultural and supporting ecosystem services (ES)^5,6^. The other side of the coin is ecosystem disservices (ED) linked to the presence and biological activity of plants in anthropogenic contexts, such as damage to property and people and pathologies triggered by plants pollen emission^7^. There is a dance between the natural and the urban environment: the expansion of urban agglomerations tends to crush green spaces, and the fragments of the natural ecosystem embedded in cities tend to their equilibrium when the energy contribution given by human management ceases. All the above results in a variation in the supply and type of ES and ED^2,3^. Provision of ES and/or ED by green urban areas depends mainly on urban forest structure and management, through which the trade-off in the provision of ES and ED can be determined^8,9^. In the dynamics of ES evaluation there is a growing need for methods and models used by the research community and the stakeholders to map and assess ES in the planning and policy process^10,11^. A local scale understanding of multiple ES and ED associated with man-made, engineered, designed natural landscapes such as urban gardens and historic parks, and proper tools for their evaluation, is lacking^9,12–15^. The proper assessment of ES and ED trade-off requires the development and the implementation of tools that can improve our ability to understand the environment, through conceptual rigor, clear rationale and objectives, transparent and easy-to-use methods, and appropriate involvement of stakeholders and practitioners^11^. To date, few researches have undertaken comprehensive assessments of the ecosystem services of urban and peri-urban forests (UPFs)^16^. Several successful tools have been developed, also open source ones^17,18^, however few studies have adopted interdisciplinary perspectives to assess the ecological, economic and cultural attributes of ecosystem services^16,19^. In this context, trade-off analysis can be a key component in integrating ES into planning and designing cities for sustainable land use^20,21^. Understanding how UPFs provide ES and ED can help avoiding the impact of human intervention that can eventually lead to drastic changes in ecosystem service provision^22^. However, many studies identify trade-offs without considering their spatial distribution^23^. Cuoeva et al.^24^ pointed out the need to include a spatial analysis in the study of ecosystem services and trade-offs to transform UPF into more resilient, healthy, and multifunctional systems.

The aim of this work is to propose a methodology useful to the integrated analysis of the simultaneous provision of ecosystem services^9^ and disservices by an urban forest through data spatialization^10,13,15^. The methods described in this study aims to configure a tool for the assessment and visualization of multiple ecosystem services and disservices in any urban forest, also highlighting the dependence of ES and ED provision on the basis of the different urban forests structure and management.

To this end, the urban forest represented by the Real Bosco di Capodimonte (RBC) (Naples, Southern Italy) has been chosen as a case study. This urban park is the biggest one in a city characterized by well-known and serious environmental issues^4^. Linked to the presence of plant cover in the urban forest area, we chose the following ES and ED to be analyzed: biodiversity, carbon storage, gross and net carbon sequestration, lessen runoff (difference between amount of surface runoff without and with tree cover), oxygen production, air pollution removal, UV effects reduction, pollen-related allergenicity risk, volatile organic compounds (VOCs) emissions. Some ES were chosen to model atmospheric and climate regulation. In addition, we chose to model avoided runoff since Naples has experienced a strong and often uncontrolled urbanization from 1943 to 2015, which grew up to 67.2% at the municipality scale^4,25^, resulting in a large number of strategic assets potentially affected by flooding^26^. Also, the pollen related ED was assessed since Naples area is well-known to be affected by important pollen issue and related human health diseases^27,28^.

In this study, 2019 was chosen as the reference year from which to draw data. In that year, the RBC was visited free of charge by 1,911,344 people^29^.

## Results

### The updated Flora of Real Bosco di Capodimonte

The first result from phytosociological survey conducted within this study was an updated Flora of the Real Bosco di Capodimonte (Supplementary Material). Our survey highlighted that 441 different plant species characterize the area, showing that 42 new species have settled in the RBC since 1992^30^. The registered species belong to 292 different genera and 100 different families. The most represented genera were *Quercus* and *Trifolium* (1.59% both), while the largest family was Asteraceae (9.07%) . The flora was arranged phylogenetically^31^ using current botanical nomenclature (POWO, n.d.) (Supplementary Material).

### Ecosystem services and disservices values

**Figure 1:**
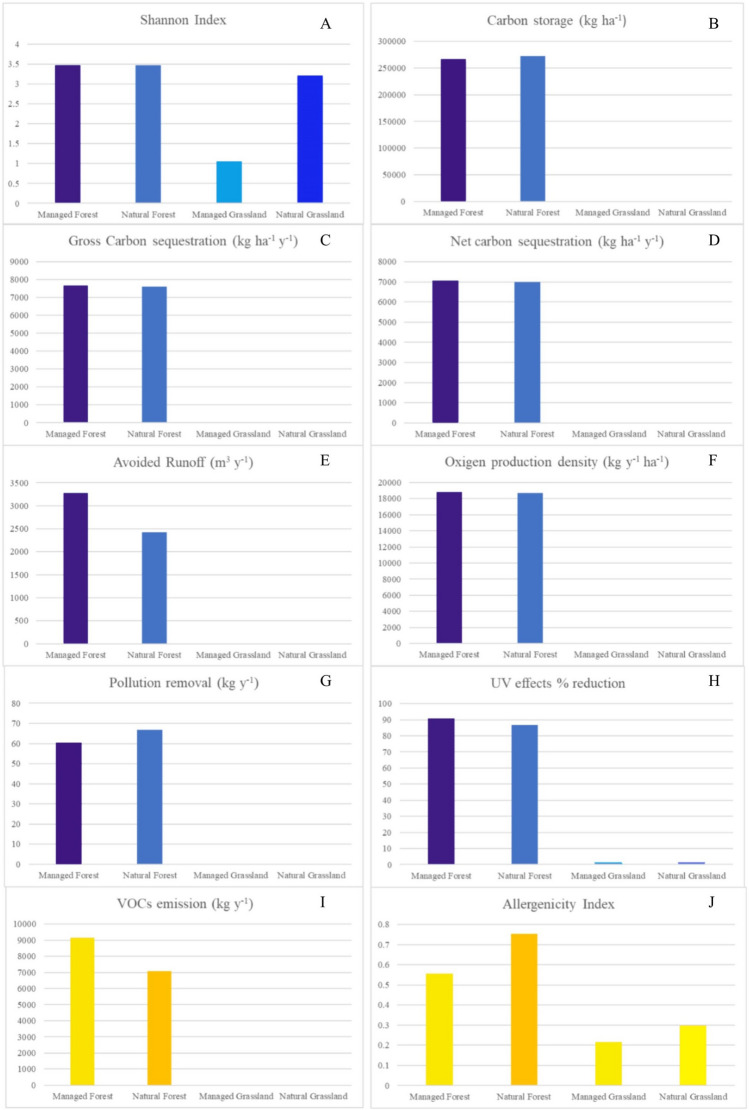
Histograms showing the comparison between managed forests, natural forests, managed grasslands and natural grasslands of the RBC in the provision of ecosystem services (blue bars, from **A**–**H**) and ecosystem disservices (yellow bars, **I** and **J**).

The total area of the RBC amounts to 132 ha. The area providing the ecosystem services and disservices investigated in this work amounts to about 106.2 ha, most of which were forests, managed (49.3 ha) and natural (38.2 ha), and the remaining part were grasslands, managed (3.9 ha) and natural (14.8 ha). The phytosociological surveys made it possible to determine the value of the specific diversity of each compartment based on the Shannon-Weiner index (H’), which was generally higher in forest areas than in grasslands (Fig. 1A). Managed forest was the compartment with the highest biodiversity value (H′ = 3.48) (Fig. 1A), and MG was the compartment with the lowest biodiversity value (H′ = 1.06) (Fig. 1A). The absolute value for the Shannon-Weiner index achieved in natural forest and natural grassland areas was 3.47 and 3.21 respectively (Fig. 1A).

Regulating ecosystem services (carbon storage, net and gross carbon sequestration, oxygen production density, pollution removal, lessen runoff) and VOCs emission related ED obtained with i-Tree Eco model, were only assessed for the forest area (both natural and managed) of the RBC due to the limitations of method used, which does not consider the contribution of non-forest areas (Fig. 1). The result of the i-Tree model carbon storage estimation in the RBC was 21.6 10^3^ Mg in total, of which 11.2 10^3^ Mg were estimated for the managed forest, and the remaining 10.4 10^3^ Mg were estimated for the natural forest. Dividing the values obtained by the model for the areas of each forest compartment, it was possible to highlight that the NF presented a carbon storage value higher than those found for the MF, 272.6 Mg ha^−1^ and 266.5 Mg ha^−1^ respectively (Fig. 1B). In contrast to the carbon storage estimate, both the calculated annual gross and net carbon sequestration data (kg ha^−1^ y^−1^) showed that these ecosystem services are provided, albeit slightly, more by the managed forest than by the natural forest (Figs. 1C,D). The gross carbon sequestration values were 7612.8 kg ha^−1^ y^−1^ for NF and 7662.9 kg ha^−1^ y^−1^ for MF, and the net sequestration ones were 6995.8 kg ha^−1^ y^−1^ for NF and 7057.86 kg ha^−1^ y^−1^ for MF (Figs. 1C,D). The Managed Forests provided an ecosystem service related to avoided runoff of 3272.65 m^3^ y^−1^, while NF provided the same for a value of 2425.17 m^3^ y^−1^ (Fig. 1E). The estimation of the ecosystem service related to oxygen production density highlighted that MF (18,820.9 kg y^−1^ ha^−1^) contributed more to the provision of this service than NF (18,665.5 kg y^−1^ ha^−1^), while it was not possible to estimate the contribution of grasslands, also in this case, due to the limitation of the model used (Fig. 1F). The i-Tree model allowed to calculate the contribution of forests to the removal of particulate and gaseous pollutants from the atmosphere (Fig. 1G), showing that, in relation to improving air quality, NF (66.75 kg y^−1^) annually contributed more than MF (60.48 kg y^−1^) (Fig. 1G). The last ES considered was the percentage reduction in the effect of UV radiation by plants. The results of the quantification of this ES showed that the greatest reduction was found in areas of MF (90.64%), followed by NF (86.73%) and grassland (1.22% for both NG and MG) (Fig. 1H). The model used allowed to obtain the quantification of the ecosystem disservice of worsening air quality due to the emission of VOCs by tree plants in forest areas (Fig. 1I). Managed forests were found to be the largest emitters of VOCs (9140.3 kg y^−1^), followed by NF (7084.9 kg y^−1^) (Fig. 1I).

The results of the modI_UGZA_ calculation showed a risks related to pollen emissions for sensitive individuals in all the land-cover and management areas except for MG, which allergenicity index value remained below the threshold value of 0.3 (Fig. 1J). The highest allergenicity index was attributed to NF (0.75), followed in the order by MF (0.56) and NF (0.30) (Fig. 1J).

### Mapping of ecosystem services, ecosystem disservices and average scores

**Figure 2:**
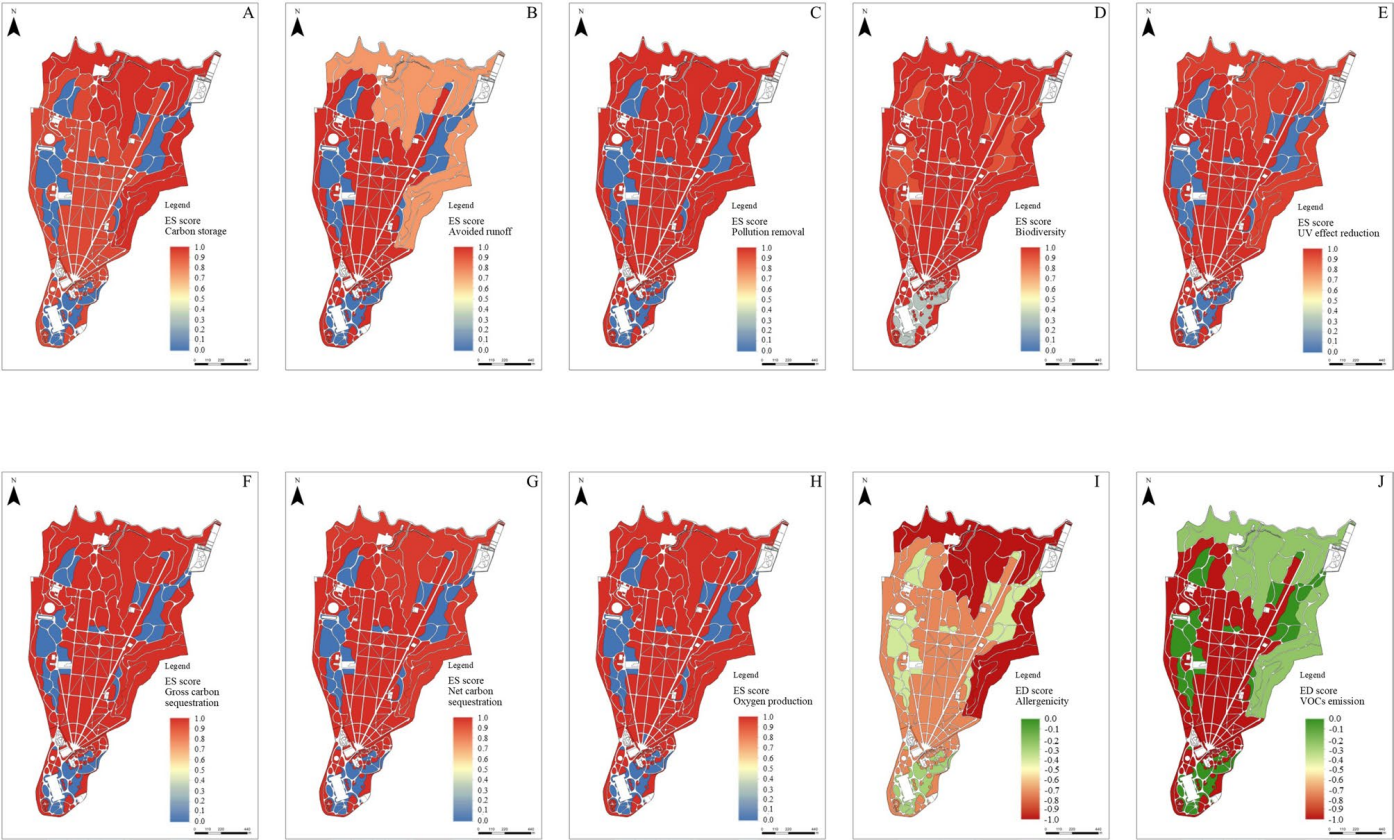
Raster maps of the RBC depicting the spatial distribution of ecosystem services and disservices in managed forests, natural forests, managed grasslands, and natural grasslands. For ecosystem services related to carbon storage (**A**), avoided runoff (**B**), pollution removal (**C**), biodiversity (**D**), UV effect reduction (**E**), gross carbon sequestration (**F**), net carbon sequestration (**G**), oxygen production (**H**), the ES score has been depicted with colorimetric scale, from 1.0 (red = maximum) to 0.0 (blue = minimum). For ecosystem disservices related to allergenicity (**I**) and VOCs emission (**J**) the ED score has been depicted with colorimetric scale, from 0.0 (green = maximum) to −1.0 (red = minimum). Raster maps have been created in QGIS environment Version 3.26.1 (http://qgis.osgeo.org) and modified in GIMP software Version 2.10.32 (https://www.gimp.org).

The values of each ES and each ED were normalized to obtain a score between 0 and 1 for ES, and 0 and −1 for ED, where 1 and −1 represented the maximum value reached for each service or disservice respectively, and 0 represented the minimum value for the scores (Table 1). The score values obtained for each of the vegetation cover and management types (MF, NF, MG, NG) were assigned to each corresponding group of polygons in the GIS environment and individual raster maps were produced for each ES or ED tested (Fig. 2).

**Table 1:** Values of ecosystem services score, ecosystem disservices score and averaged ES–ED score estimated for managed forests (MF), natural forests (NF), managed grasslands (MG) and natural grasslands (NG) of the RBC.

	Shannon Index	Carbon storage	Gross Carbon sequestration	Net Carbon sequestration	Avoided Runoff	Oxigen production density	Pollution removal	UV effects% reduction	VOCs emission	Allergenicity Index	Average score
	ES								ED		ES–ED
MF	1.000	0.978	1.000	1.000	1.000	1.000	0.906	1.000	−1.000	−0.740	0.614
NF	0.999	1.000	0.993	0.991	0.741	0.992	1.000	0.957	−0.775	−1.000	0.590
MG	0.305	0.000	0.000	0.000	0.000	0.000	0.000	0.013	0.000	−0.287	0.003
NG	0.924	0.000	0.000	0.000	0.000	0.000	0.000	0.013	0.000	−0.396	0.054

**Figure 3:**
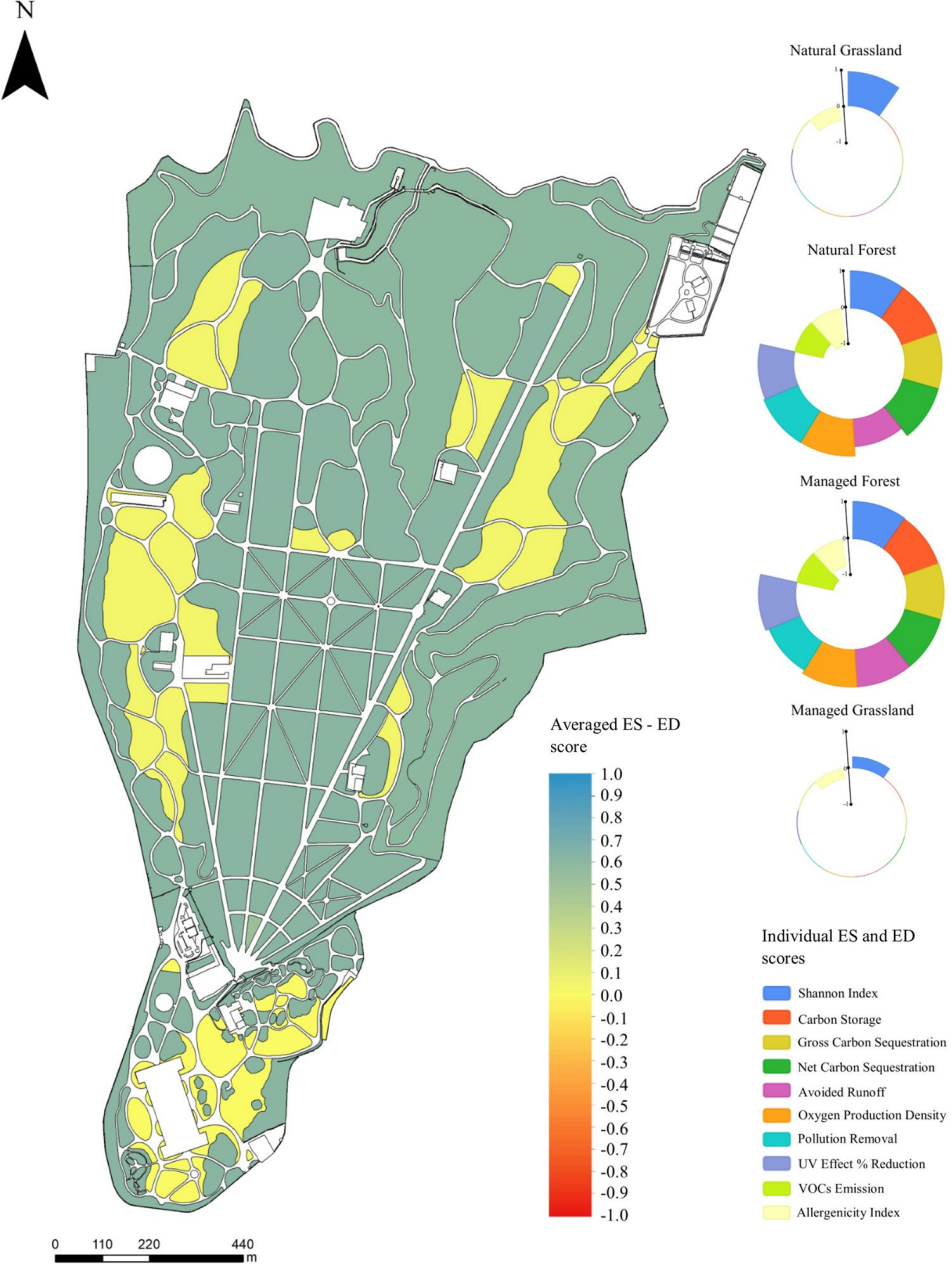
Raster map of the average score value reached in managed forest, natural forest, managed grassland and natural grassland of RBC. The color scale used ranges from a maximum value of 1 (blue) to a minimum value of −1 (red). On the right are circular barplots for each area with different vegetation cover and management highlighting the contribution of the individual ES and ED scores (Table 1) obtained by standardizing the values estimated from the analyses. Raster map has been created in QGIS environment Version 3.26.1 (http://qgis.osgeo.org) and modified in GIMP software Version 2.10.32 (https://www.gimp.org).

The standardized ES and ED score values (Table 1) allowed us to work mathematically in a GIS environment to determine and map the averaged ES–ED score taking into account both the contribution of ecosystem services and that of ecosystem services (Fig. 2). The highest averaged ES–ED score was obtained by the MF, followed by the NF (Table 1). Among grasslands, the sector with the highest averaged ES–ED score was NG, while in absolute terms the lowest value was for MG (Table 1). The averaged ES–ED scores were used to realize a raster map which made it possible to discriminate the difference in the concomitant provision of ES and ED between the compartments with different vegetation cover (forest or grassland), but does not show differences between the compartments with the same vegetation cover and different management (Fig. 3).

## Discussion

### RBC ecosystem services and ecosystem disservices

In this work, we evaluated and quantified some of the ES and ED provided by the urban park Real Bosco di Capodimonte. The main aims were mapping their provision in the different plant cover compartments under different management (managed or not managed) and assess a methodology useful to simultaneously evaluate the trade-off considering both ecosystem services and disservices in an urban park^9,10^.

The RBC areas showing the highest averaged ES–ED score were those belonging to the MF category, in which, considering the contribution of ecosystem services and disservices, an averaged ES–ED score value of 0.614 was calculated (Table 1 and Fig. 3). The better performance of this land cover and management category over the remaining three could be found in ecosystem services related to biodiversity, carbon sequestration (gross and net), reduction of runoff and UV effects, and oxygen production (Table 1 and Fig. 2), despite the fact that MF also showed the highest score of ED related to the VOCs emission (Table 1 and Fig. 2J).

The value of biodiversity were slightly higher in MF than in the NF in RBC. This was peculiar as other studies highlighted that areas with greater naturalness have greater specific richness, and that these natural areas were usually managed less to leave the natural evolutionary course of the potential vegetation unaltered^9,32,33^. On the other hand, in a historic park, value of biodiversity could tend to be greater in the managed areas because targeted management allows the preservation of exogenous, non-invasive elements (e.g. exotic specimens), as well as those senescent and centuries-old individuals that would more easily meet their death in the absence of management^34,35^. Management also makes it possible to promote natural endemic species by controlling allochthonous and invasive species^36^. For grasslands, an increase in biodiversity is observed in natural rather than managed settings (Table 1, Fig. 2D). Natural grasslands turn out to be a remarkable reservoir of biodiversity^37^, while human intervention flattens diversity favoring the provision of different ES, such as recreational services^38^. Results showed that forest and grassland management influences the value attributable to biodiversity-related ES (Table 1, Fig. 2D). Specifically, two divergent trends can be noted: MF show a higher value than NF, while grasslands follow the opposite logic (Table 1, Fig. 3).

The carbon stock results obtained in the RBC were in line with results obtained for other historical park in the Mediterranean area^9^. In the RBC, MF and NF attested a carbon stock value ranging from 272 Mg ha^−1^ to 266 Mg ha^−1^, exceeding the carbon stock European average (162 Mg ha^−1^) estimated by^39^ and what has been measured in a case study in Finland^40^. The study conducted in Helsinki urban park showed that the carbon stock in trees depends mainly on the age of plant formation^40^.The case study presented in this paper is characterised by the massive presence of tree individuals over three centuries old, which combined with the density of tree formations, (that have been maintained in their original spatial conformation) justifies the estimated carbon stock in forest areas^9,42^.

Gross and net carbon sequestration was both higher in the MF than in the NF (Table 1, Fig. 2F,G). The i-Tree model was also been used to estimate the value of these ES^17,41–43^. Comparing the provision of this ES between two different contexts, albeit belonging to the same typology of green structures, could be tricky due to differences in calculation or estimation methodology^9^. The result of the analysis carried out in this case study showed a value for gross carbon sequestration of 668.6 Mg y^−1^ and for net carbon sequestration of 615.2 Mg y^−1^, which distributed over the surface (ha^−1^) of the different areas of the park, resulted in the values reported in this work.

Natural Forests mainly provided air quality related ES, while MF provided highest ES related to oxygen production and reducing surface runoff (Fig. 2). As with other examples in the literature, the best performance in terms of pollutant sequestration can be attributed to structures characterised by a higher canopy density^44–46^. In this sense, management aimed at canopy density limitation (for safety reason) could depress the provision of these ES.

The calculation of the allergenicity ecosystem disservice through the modI_UGZA_ model resulted in the absence of ED only for MG, since the allergenicity thresholds were not reached. Of the estimated allergenicity indices, the one for NG is equal to the threshold limit (0.30), and in NF and MF values exceeded the threshold indicating the risk of triggering pollen diseases in sensitive individuals (Table 1)^7,47^. In agreement with other authors, the increase in allergenicity can be attributed to the increased presence of highly allergenic species (e.g. Poaceae), as in the case of natural grasslands, or to the non-managed tree compartments^7,47^.

The creation of raster maps in a GIS environment allowed a clear view of how the single ES or ED is distributed in the urban park area according to plant cover and management, and how the intensity of the value associated to them varied (Fig. 2). It also made possible, through the calculation of averaged ES–ED scores, to explore the trade-off between the ecosystem services and disservices provided in the various areas of the park using a methodology that is easy to apply in any context, even changing the services (or disservices) types as well as the tools used to provide raw data (Fig. 3). The results showed that, in this case study, the dominant structure of the landscape compartment (forest or grassland) mostly influences and regulates the provision of single ES and ED rather than the type of management to which it is subject. On the other hand, management becomes the most driving factor when considering the same type of landscape compartment (Table 1, Fig. 3)^48^.

### Limitations and perspectives

In this section, the main limitations are also considered. The used model has some issues in data acquisition and processing^49,50^, such as the inability to evaluate the contribution of grassland layer in ES and ED provision^17^. This represents a limitation in the estimation of the average score of ecosystem services provided by the RBC, due to underestimation. Studies supported this assertion attesting the role of grasslands in carbon sequestration and regulation of the carbon cycle^51,52^, and in the removal of air pollutants^53^. Despite this obvious limitation in calculating the grassland averaged ES–ED score, i-Tree Eco model has been used both for the simplicity of its application and because it enabled to obtain many useful data^17^. Furthermore, recent studies have compared the performance of the i-Tree Eco model with eddy covariance technology and obtained similar results in estimating pollutant removal by canopy^54^. Moreover, i-Tree Eco model has been used in many studies at international level, both purely application-oriented and for research purposes^17,49,50^.

Information obtained from the analysis of an individual ES or one ED can only partially provide an accurate picture of the actual value of an urban park. However, the simultaneous assessment of multiple ES and ED, and the identification of trade-offs performed through the proposed averaged ES–ED scores, could provide an overview of the real value of a urban park and identify the main drivers useful to maximize the delivery of ES, limiting the delivery of ED where possible^9,22,55^. Urban ecosystem management thus acquires considerable importance in maintaining a positive balance in the provision of ecosystem services^56^.

## Conclusion

The Real Bosco di Capodimonte is a complex natural ecosystem, inserted in an extremely urbanized context represented by the city of Naples. The methodology presented in this article provided a clear view of some of the ecosystem services and disservices that the park constantly provides, and the existing relationship between them, the vegetation structure and the management. The management of the park makes it possible to increase or maintain the supply of ES and to limit what could be ED, as well as to regulate their distribution from a spatial point of view. The product of this work could certainly represent a useful methodology for increasing the awareness of managers and decision-makers of green urban areas, as well as a tool for monitoring and simulating the variation of ecosystem service provision as management practices or environmental conditions change^10,57^.

In conclusion, we highlighted the limitations and potential of the proposed methodology. Due to its constitution, the methodology lends itself to being extended with any type of ES or ED, and could be also applied in any context of urban green areas after knowing the structure of the system. Moreover, it could be used as a tool for the targeted management of urban green areas and for the dissemination of scientific data, which are not always easily understood by the population.

## Materials and methods

### Study area

**Figure 4:**
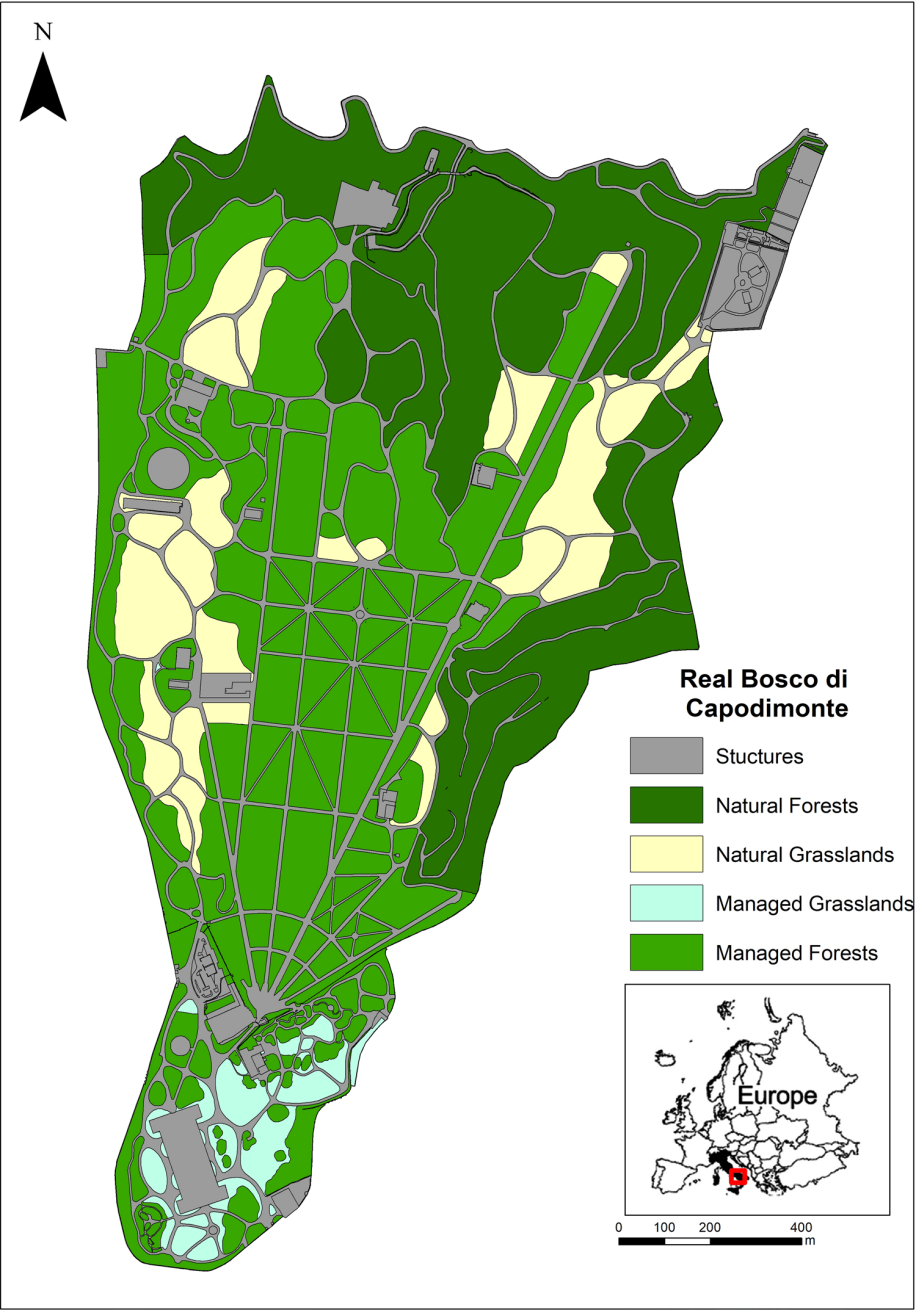
Map of the Real Bosco di Capodimonte (Naples, Southern Italy) with details of forest and grassland cover by management type. Map has been created in QGIS environment Version 3.26.1 (http://qgis.osgeo.org).

The chosen urban forest was the Real Bosco di Capodimonte (40.8725° N, 14.2533° E), an urban public park located in Naples (southern Italy), which is one of the largest in the Country, extending over 132 ha^58^, and annually visited by ca. 3,000,000 people. The park was established in 1734 by the Bourbon dynasty and began as a hunting reserve configured as a fully planted holm oak (*Quercus ilex* L.) grove. Over the following centuries, the structure of the park was profoundly modified and large areas of grassland were created following the removal of part of the tree cover^59^. The succession of changes led to the enrichment of the plant landscape through the introduction of exotic and rare tree species, many of which still exist in the park^30^. A floristic study carried out by La Valva et al.^30^ revealed the presence of 399 plant species, in 108 families and 274 genera, in the RBC. Today, the RBC is a complex landscape garden composed of forest (87.5 ha) and grassland (18.7 ha), while the remaining area is made up of buildings, structures, avenues and paths through the park. The vegetation cover present can be distinguished based on the type of management applied in two categories for each type of formation. The forest areas can be separated into "Managed Forests" (MF) and "Natural Forests" (NF), and the grassland can be separated into “Managed Grassland” (MG) and “Natural Grassland” (NG) (Fig. 4).

**Figure 5:**
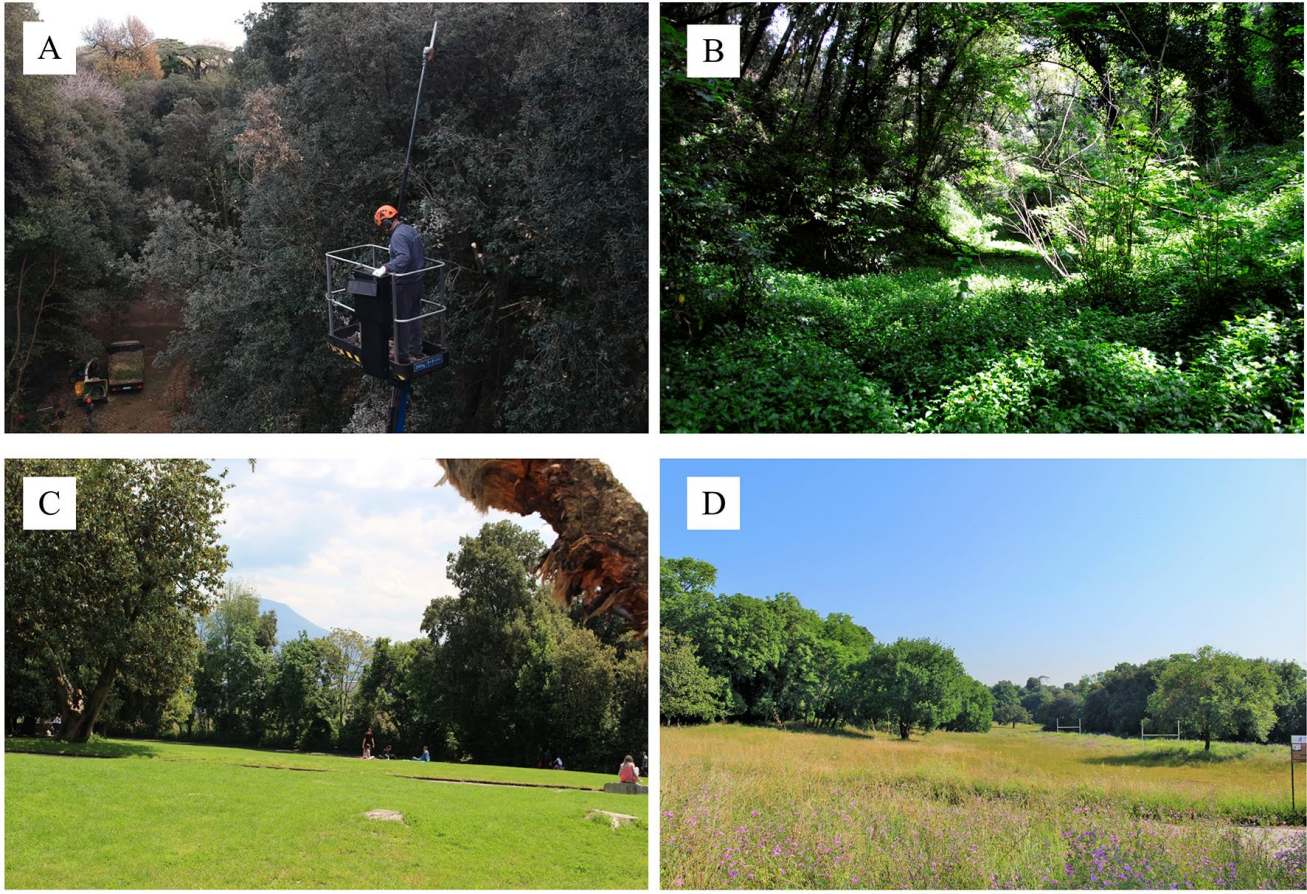
Plant cover compartments under different management type of the RBC (Naples, Southern Italy): Managed Forests (**A**), Natural Forests (**B**), Managed Grasslands (**C**) and Natural Grasslands (**D**).

Managed Forests, covering about 49.3 hectares, are actively managed through daily recognition of forested areas, routine maintenance operations on a weekly basis, and non-routine maintenance operations. Mainly routine management operations include silvicultural operations for undergrowth containment and eradication of invasive alien species, both herbaceous and tree. Non-routine maintenance is mainly carried out to ensure the safety of visitors through Visual Tree Assessment (VTA), which may be followed by pruning for canopy lightening, cutting of individuals that pose safety problems, and removal of woody material (Fig. 5A). These operations are accompanied by replanting of trees in order to keep the historic forest system stable and safe. Natural Forests cover 38.2 ha and is characterized by the total absence of any type of management, due to the orography of the territory and the difficulty of access, which has led to a forest formation tending towards the potential vegetation of the area and the introduction of allochthonous and invasive forest species (Fig. 5B). As for grasslands, MG occupies 3.9 hectares and consists of heavily managed herbaceous covers in which mowing is carried out weekly, irrigation in the summer period twice a day, fertilization when necessary (Fig. 5C). The most important interventions in these areas are periodic turf eradication and subsequent sowing of selected species (mainly Graminaceae and Fabaceae) to obtain a lawn useful for the recreational purposes of park visitors (Fig. 5C). On the other hand, NG (14.8 ha) consists of grasslands that are not affected by any active management except for summer cutting to reduce fire risk (Fig. 5D). Mowing in these areas is done only after the phenological stage of flowering and fruiting of component species, especially the valuable native Orchidaceae, so as to preserve biodiversity in the meadows.

### Phytosociological survey, Capodimonte Flora update and Biodiversity assessment

The entire area of the RBC has been mapped in a GIS environment (“QGIS. Geographic Information System. QGIS Association. 2021,” n.d.) (Fig. 4) and, in each cover and management type, floristic surveys have been carried out in 2019 in order to collect data useful for the determination and quantification of the related ecosystem services and disservices. In proportion to the size of each landscape compartment, a different number of 400 m^2^ square-shaped sampling areas were randomly localized and materialized within which the plant species present were sampled and identified using dichotomous keys^60^. The area covered by each species was estimated using the Braun-Blanquet methodology^61^ which is widely used to assess the cover-abundance values^17,62,63^. The information contained in the Flora of the RBC made in La Valva et al.^30^ was updated and implemented through the phytosociological survey.

The updated flora resulting from the field operations was compiled using the botanical nomenclature currently in use, drawing information from online databases^64^, and sorted by APG IV phylogenic criteria^31^. Phytosociological surveys of the sampling areas provided information on the specific diversity and relative abundances of the species present in the four different landscape compartments. These data were used to determine the Shannon–Wiener diversity index (H’) for the two types of forest and grassland by combining the species richness (the number of species in a given area) and their relative abundances (converting the values obtained with the Braun-Blanquet scale)^65,66^.

### i-Tree Eco model

The i-Tree Eco model was used to determine the values of carbon storage, gross and net carbon sequestration, avoided runoff, oxygen production, air pollution removal, and VOCs emission^17,43,44^. In accordance with the model's user manual^17^, 52 sampling areas of 400 m^2^ were determined to carry out both morphological and structural analyses of the tree component. In managed forests, natural forests, managed grasslands and natural grasslands, 24, 19, 3 and 6 plot areas have been respectively materialized according to the surfaces proportions. These sampling areas was also used for phytosociological surveys. The parameters investigated and collected in field were: land use, tree species, tree stem diameter at 1.3 m from ground, tree total and crown height, tree crown width (N–S and E–W), number of tree crown side exposed to the sunlight, tree crown missing and dieback, percentage of land covered by shrubs, shrub species, shrub height^17^. The climate and air quality data from the Campania region (2019) air quality monitoring system database were used by the model and the technical report was produced after the data were submitted.

### Pollen-related disservice assessment

The evaluation and quantification of the ED linked to the risk of allergic reactions caused by the emission of pollen from plants present in the RBC was carried out by calculating the Index of Urban Green Zones Allergenicity (I_UGZA_)^7^. The index is calculated by taking into account the vegetal species of the area and associating each species with the potential allergenic value (VPA), linked to species-specific biological parameters (allergenic potential, principal pollination period, pollen emission), and the volume occupied by the specimens belonging to the species (plant height * canopy projection)^7^. In this work, due to the size of the park under study, the methodology proposed by^28^ (*modI*_*UGZA*_) was applied, which involves determining the volume occupied by the species by means of an area estimation made with the Braun-Blanquet scale^61^. For each of the landscape compartments the formula (Eq. 1) was applied where: *modI*_*UGZA*_ is the modified index, *maxH* is the maximum height between the species in the landscape compartment, *maxVPA* is the maximum potential allergenic value between the species found, *S*_*c*_ is the area of the considered landscape compartment, *k* is the number of species found, *VPA*_*i*_, *SBB*_*i*_ and *H*_*i*_ are the potential allergenic value, the occupied surface averaged with Braun-Blanquet scale, and the average height of the *i*-th species found, respectively.

1 $${modI}_{UGZA}= \frac{1}{maxH*maxVPA*{S}_{c}}\sum_{i=1}^{k}{VPA}_{i}*{SBB}_{i}* {H}_{i}$$

Equation 1—formula for the modI_UGZA_ assessment (Prigioniero et al.^49^).

In order to apply the formula, data from the phytosociological survey were used, while bibliographic sources^7,60,67^ were used to determine the VPA and the average heights of the species. I_UGZA_ (and its modified version *modI*_*UGZA*_) values can range from 0 to 1, and the threshold risk of triggering allergic reactions caused by pollens is 0.3^68,69^.

### Data analysis and spatial mapping

The values of each considered ES and ED was assessed independently in each vegetation and management area of the park (natural and managed forests and grasslands). Following the methodology described in^9^, to compare the contribution to the value of ES and ED, we (i) assumed equal importance for each ES and ED, (ii) standardized the values of ES and ED weighting by the highest value obtained for that service or disservice in the RBC, so as to obtain an ES and ED score between 0 and 1 (where 1 was the maximum value), (iii) convert ED scores in negative values, (iv) calculated the average between the scores of ES and ED assessed in each vegetation and management area, defined as “Averaged ES–ED score” (Fig. 3). For mapping purposes, each ES and ED score values were assigned to each polygon of MF, NF, MG and NG. Thereafter, each layer were converted to raster choosing as value the ES and ED score and a proper cell size of 1 m^2^. After obtaining the individual ES and ED rasters, the Raster Calculator tool in QGIS environment was used to average the individual rasters and obtain the Averaged ES–ED score of the RBC. The Raster Calculator allows performing calculations on the basis of existing raster pixel values and results are written to a new raster layer.

## Data availability

All data generated or analysed during this study are included in this published article [and its supplementary information files].

## Supplementary Information

Supplementary Information.
