# Supplementary material for: Holistic tool for ecosystem services and disservices assessment in the urban forests of the Real Bosco di Capodimonte, Naples
Source: Sci Rep. 2022 Sep 30;12:16413. doi: 10.1038/s41598-022-20992-0 (PMC9525253; doi:10.1038/s41598-022-20992-0)
Supplement: Supplementary file 1 — Supplementary Information. [file 41598_2022_20992_MOESM1_ESM.docx]

Supplementary Material

Holistic tool for ecosystem services and disservices assessment in the urban forests of the Real Bosco di Capodimonte, Naples

Flora of Real Bosco di Capodimonte

Updated Flora of the Real Bosco di Capodimonte sorted phylogenetically with APG IV criteria using current botanical nomenclature from Kew Botanical Garden (Plant Of the World Online) database.

**Legend:** ORDER**; Family;** Genus; *Species*

## POLYPODIALES

### Dennstaedtiaceae

Pteridium

*Pteridium aquilinum* (L.) Kuhn subsp. *aquilinum*

### Pteridaceae

Adiantum

*Adiantum capillus-veneris* L.

Anogramma

*Anogramma leptophylla* (L.) Link

### Dryopteridaceae

Cyrtomium

*Cyrtomium falcatum* (L. fil.) C. Presl subsp. *falcatum*

Polystichum

*Polystichum aculeatum* (L.) Roth.

### Polypodiceae

Polypodium

*Polypodium cambricum* subsp. *cambricum*

### Aspleniaceae

Asplenium

*Asplenium adiantum-nigrum* L.

*Asplenium onopteris* L.

*Asplenium scolopendrium* L. subsp. *scolopendrium*

*Asplenium trichomanes* L.

## CASUARINALES

### Casuarinaceae

Casuarina

*Casuarina equisetifolia* L.

## Cycadales

### Cycadaceae

Cycas

*Cycas revoluta* Thunb.

## Gingkoales

### Gyngkoaceae

Gingko

*Ginkgo biloba* L.

## Pinales

### Aracauriaceae

Araucaria

*Araucaria angustifolia* (Bertol.) Kuntze

### Podocarpaceae

Podocarpus

*Podocarpus elongatus* (Aiton) L'Herit. ex Pers.

### Pinaceae

Abies

*Abies nordmanniana* (Steven) Spach

*Abies pinsapo* Boiss.

Cedrus

*Cedrus atlantica* (Endl.) Manetti ex Carriere

*Cedrus deodara* (Lamb.) G. Don

*Cedrus libani* A. Rich.

Pinus

*Pinus halepensis* Mill.

*Pinus nigra* J.F. Arnold

*Pinus pinaster* Aiton

*Pinus pinea* L.

### Cephalotaxaceae

Cephalotaxus

*Cephalotaxus harringtonii* (Knight ex J. Forbes) K. Koch var. *harringtonii*

### Taxaceae

Taxus

*Taxus baccata* L.

### Cupressaceae

Cupressus

*Cupressus sempervirens* L.

Platycladus

*Platycladus orientalis* (L.) Franco

Taxodium

*Taxodium distichum* (L.) Rich.

*Taxodium mucronatum* Ten.

## Piperales

### Aristolochiaceae

Aristolochia

*Aristolochia sempervirens* L.

## Laurales

### Lauraceae

Cinnamomum

*Cinnamomum camphora* (L.) J. Presl

Laurus

*Laurus nobilis* L.

## Magnoliales

### Magnoliaceae

Liriodendron

*Liriodendron tulipifera* L.

Magnolia

*Magnolia grandiflora* L.

*Magnolia obovata* Thunb.

*Magnolia × soulangeana* Soul.-Bod.

*Magnolia kobus* DC.

## Alismatales

### Araceae

Arisarum

*Arisarum vulgare* O.Targ.Tozz.

Arum

*Arum italicum* Miller subsp. *italicum*

## Dioscoreales

### Dioscoreaceae

Dioscorea

*Dioscorea communis* (L.) Caddick & Wilkin

## Liliales

### Smilacaceae

Smilax

*Smilax aspera* L.

## Asparagales

### Amaryllidaceae

Allium

*Allium neapolitanum* Cirillo

*Allium vineale* L.

Agapanthus

*Agapanthus africanus* (L.) Hoffmanns.

Narcissus

*Narcissus tazetta* L.

### Asparagaceae

Agave

*Agave americana* L.

Asparagus

*Asparagus officinalis* L.

*Asparagus acutifolius* L.

Liriope

*Liriope muscari* L.

Ornithogalum

*Ornithogalum exscapum* Ten.

Ruscus

*Ruscus aculeat*us L.

*Ruscus hypoglossum* L.

### Iridaceae

Iris

*Iris foetidissima* L.

### Orchidaceae

Cephalanthera

*Cephalanthera longifolia* (L.) Fritsch

Seriapias

*Serapias vomeracea* (Burm.f.) Briq.

## Arecales

### Arecaceae

Butia

*Butia capitata* (Mart.) Becc.

Chamaerops

*Chamaerops humilis* L.

Brahea

*Brahea armata* S.Watson

Livistona

*Livistona australis* (R. Br.) Mart.

*Livistona chinensis* (Jacq.) R.Br. ex Mart.

Phoenix

*Phoenix canariensis* H.Wildpret

*Phoenix dactylifera* L.

*Phoenix reclinata* Jacq.

*Phoenix theophrasti* Greuter

Washingtonia

*Washingtonia filifera* (Rafarin) H.Wendl. ex de Bary

*Washingtonia robusta* H.Wendl.

## Poales

### Cyperaceae

Carex

Carex divulsa Stokes

*Carex flacca* Schreber subsp. *flacca*

*Carex pendula*Huds.

Cyperus

*Cyperus rotundus* L.

### Juncaceae

Luzula

*Luzula forsteri* (Sm.) DC.

### Poaceae

Achnatherum

*Achnatherum miliaceum* (L.) P.Beauv.

Agrostis

*Agrostis stolonifera* L.

Anthoxanthum

*Anthoxanthum aristatum* Boiss.

Arundo

*Arundo donax* L.

Avena

*Avena barbata* Pott ex Link

Brachypodium

*Brachypodium sylvaticum* (Huds.) P.Beauv.

Briza

*Briza maxima* L.

Bromus

*Bromus hordeaceus* L.

*Bromus sterilis* L.

Catapodium

*Catapodium rigidum* (L.) C.E.Hubb.

Cynodon

*Cynodon dactylon* (L.) Pers.

Cynosurus

*Cynosurus echinatus* L.

Dactylis

*Dactylis glomerata* L. subsp. *hispanica* (Roth) Nyman

Echinochloa

*Echinochloa crus-galli* (L.) P.Beauv.

Eragrostis

*Eragrostis pilosa* (L.) P.Beauv.

Phalaris

*Phalaris arundinacea* L.

Holcus

*Holcus lanatus* L.

Hordeum

*Hordeum murinum* L.

*Hordeum murinum* subsp. *leporinum* (Link) Arcang.

Lolium

*Lolium perenne* L.

Poa

*Poa annua* L.

*Poa pratensis* L.

*Poa trivialis* L.

Rostraria

*Rostraria cristata* (L.) Tzvelev

Setaria

*Setaria verticillata* (L.) P.Beauv.

Sorghum

*Sorghum bicolor* (L.) Moench

Vulpia

*Vulpia ligustica* (All.) Link

*Vulpia myuros* (L.) C.C.Gmel.

## Commelinales

### Commelinaceae

Tradescantia

*Tradescantia fluminensis* Vell.

## Ranunculales

### Berberidaceae

Berberis

*Berberis aquifolium* Pursh

Mahonia

*Mahonia* x *media* Brickell

Nandina

*Nandina domestica* Thunb.

### Menispermaceae

Cocculus

*Cocculus laurifolius* DC.

### Papaveraceae

Chelidonium

*Chelidonium majus* L.

Fumaria

*Fumaria capreolata* L.

*Fumaria officinalis* L. subsp. *officinalis*

Papaver

*Papaver rhoeas* L. subsp. *rhoeas*

### Ranunculaceae

Anemone

*Anemone apennina* L.

Clematis

*Clematis vitalba* L.

Delphinium

*Delphinium halteratum* Sm.

Ficaria

*Ficaria verna* subsp. *ficariiformis* (Rouy & Foucaud) Maire

Ranunculus

*Ranunculus neapolitanus* Tenore

*Ranunculus sardous* Cr.

## Proteales

### Proteaceae

Banksia

*Banksia ericifolia* L. fil.

Grevillea

*Grevillea robusta* A.Cunn. ex R.Br.

Hakea

*Hakea laurina* R. Br.

### Platanaceae

Platanus

*Platanus hybrida* Brot.

*Platanus orientalis* L.

## Buxales

### Buxaceae

Buxus

*Buxus balearica* Lam.

*Buxus sempervirens* L.

## Saxifragales

### Crassulaceae

Sedum

*Sedum cepaea* L.

Umbilicus

*Umbilicus rupestris* (Salisb.) Dandy

## Vitales

### Vitaceae

Parthenocissus

*Parthenocissus quinquefolia* (L.) Planchon

## Oxalidales

### Oxalidaceae

Oxalis

*Oxalis corniculata* L.

*Oxalis dillenii* Jacq.

*Oxalis fontana* Bunge

## Celastrales

### Celastraceae

Euonymus

*Euonymus europaeus* L.

*Euonymus japonicus* Thunb.

## Malpighiales

### Euphorbiaceae

Euphorbia

*Euphorbia amygdaloides* L. subsp. *amygdaloides*

*Euphorbia helioscopia* L.

*Euphorbia peplus* L.

### Hypericaceae

Hypericum

*Hypericum androsaemum* L.

*Hypericum hircinum* L.

*Hypericum inodorum* Miller

*Hypericum perforatum* subsp. *veronense* (Schrank) A. Fröhlich

*Hypericum tetrapterum* Fries

### Linaceae

Linum

*Linum perenne* L.

### Euphorbiaceae

Mercurialis

*Mercurialis annua* L.

*Mercurialis perennis* L.

### Salicaceae

Salix

*Salix babylonica* L.

*Salix caprea* L.

### Violaceae

Viola

*Viola alba* Besser

*Viola arvensis* Murray

*Viola odorata* L.

*Viola reichenbachiana* Jord. ex Boreau

## Fabales

### Fabaceae

Acacia

*Acacia dealbata* Link

Albizia

*Albizia julibrissin* Durazz.

Bauhinia

*Bauhinia aculeata* subsp. *grandiflora* (Juss.) Wunderlin

Bituminaria

*Bituminaria bituminosa* (L.)C.H.Stirt.

Calliandra

*Calliandra tweedii* Benth.

Ceratonia

*Ceratonia siliqua* L.

Cercis

*Cercis siliquastrum* L.

Cytisus

*Cytisus villosus* Pourr.

Dorycnium

*Dorycnium hirsutum* (L.)Ser.

Hippocrepis

*Hippocrepis emerus* subsp. *emeroides* (Boiss. & Spruner)Lassen

Lotus

*Lotus corniculatus* L.

*Lotus edulis* L.

Medicago

*Medicago lupulina* L.

*Medicago murex* Willd.

*Medicago rigidula* (L.)All.

*Medicago sativa* L.

Melilotus

*Melilotus albus* Medik.

Ornithopus

*Ornithopus compressus* L.

Robinia

*Robinia pseudoacacia* L.

Trifolium

*Trifolium arvense* L.

*Trifolium campestre* Schreb.

*Trifolium dubium* Sibth.

*Trifolium nigrescens* Viv. subsp. *nigrescens*

*Trifolium patens* Schreb.

*Trifolium pratense* L. var. *pratense*

*Trifolium repens* L. var. *repens*

Vicia

*Vicia villosa* Roth subsp. *ambigua* (Guss.)Kerguelen

## Rosales

### Cannabaceae

Celtis

Celtis australis L.

### Elaeagnaceae

Elaeagnus

*Elaeagnus umbellata* C. P. Thunb. ex A. Murray

*Elaeagnus* × *submacrophylla* Servett.

### Moraceae

Ficus

*Ficus carica* L.

### Rhamnaceae

Paliurus

*Paliurus spina-christi* Miller

### Rosaceae

Agrimonia

*Agrimonia eupatoria* L.

Aphanes

*Aphanes microcarpa* (Boiss. & Reut.) Rothm.

Crataegus

*Crataegus monogyna* Jacq.

Eriobotrya

*Eriobotrya japonica* (Thunb.) Lindl.

Geum

*Geum urbanum* L.

Mespilus

*Mespilus germanica* L.

Potentilla

*Potentilla reptans* L.

Poterium

*Poterium sanguisorba* L. subsp. *sanguisorba*

Prunus

*Prunus cerasifera* Ehrh.

*Prunus laurocerasus* L.

*Prunus persica* (L.) Stokes

Pyrus

*Pyrus communis* L.

Rosa

*Rosa* sp.

Rubus

*Rubus ulmifolius* Schott

Sorbus

*Sorbus aucuparia* L. subsp. *aucuparia*

Spiraea

*Spiraea cantoniensis* Lour.

*Spiraea japonica* L. fil.

### Ulmaceae

Ulmus

*Ulmus glabra* Huds.

*Ulmus minor* Mill.

### Urticaceae

Parietaria

*Parietaria judaica* L. subsp. *judaica*

*Parietaria lusitanica* L.

Urtica

*Urtica dioica* L.

*Urtica membranacea* Poir.

## Cucurbitales

### Cucurbitaceae

Bryonia

*Bryonia cretica* L. subsp. *cretica*

## Fagales

### Betulaceae

Alnus

*Alnus cordata* (Loisel.) Duby

Carpinus

*Carpinus betulus* L.

*Carpinus orientalis* Mill.

Corylus

*Corylus avellana* L.

Ostrya

*Ostrya carpinifolia* Scop.

### Fagaceae

Castanea

*Castanea sativa* Mill.

Quercus

*Quercus cerris* L.

*Quercus ilex* L.

*Quercus petraea* (Matt.) Liebl.

*Quercus pubescens* Willd.

*Quercus robur* L.

*Quercus rubra* L.

*Quercus suber* L.

## Geraniales

### Geraniaceae

Erodium

Erodium botrys (Cav.) Bertol.

Geranium

*Geranium molle* L.

*Geranium purpureum* Vill.

*Geranium rotundifolium* L.

## Myrtales

### Lythraceae

Lagerstroemia

*Lagerstroemia indica* L.

### Myrtaceae

Callistemon

*Callistemon linearis* (Schrad. & Wendl.) Colv. ex Sweet

Eucalyptus

*Eucalyptus camaldulensis*Dehnh

*Eucalyptus robusta* Sm.

*Eucalyptus gunnii* Hook.f.

Melaleuca

*Melaleuca styphelioides* Sm.

Myrtus

*Myrtus communis* L. subsp. *communis*

### Onagraceae

Circea

*Circaea lutetiana* L.

Epilobium

*Epilobium lanceolatum* Sebastiani & Mauri

## Crossosomatales

### Staphyleaceae

Staphylea

*Staphylea pinnata* L.

## Sapindales

### Anacardiaceae

Cotinus

*Cotinus coggygria* Scop.

Pistacia

*Pistacia terebinthus* L.

### Rutaceae

Citrus

*Citrus aurantium* L.

*Citrus limon* (L.) Burm. fil.

*Citrus reticulata* Blanco

Ruta

*Ruta chalepensisi* L.

### Sapindaceae

Acer

*Acer campestre* L.

*Acer monspessulanum* L.

*Acer negundo* L.

*Acer opalus* subsp. *obtusatum* (Waldst. & Kit. ex Willd.) Gams

*Acer platanoides* L.

*Acer pseudoplatanus* L.

Aesculus

*Aesculus* x *carnea*

*Aesculus hippocastanum* L.

### Simaroubaceae

Ailanthus

*Ailanthus altissima* (Miller) Swingle

## Malvales

### Bombacaceae

Ceiba

*Ceiba speciosa* (A.St.-Hil.) Ravenna

### Malvaceae

Alcea

*Alcea rosea* L.

Brachychiton

*Brachychiton acerifolius* (A.Cunn. ex G.Don) F.Muell

*Brachychiton populneus* (Schott & Endl.) R.Br.

Tilia

*Tilia americana* L.

*Tilia cordata* Mill.

*Tilia platyphyllos* Scop. subsp. *platyphyllos*

*Tilia tomentosa* Moench

### Thymelaeaceae

Daphne

*Daphne laureola* L.

## Brassicales

### Brassicaceae

Alliaria

*Alliaria petiolata* (M.Bieb.) Cavara & Grande

Brassica

*Brassica fruticulosa* Cirillo

Capsella

*Capsella bursa-pastoris* (L.) Medik.

Cardamine

*Cardamine hirsuta* L.

Lepidum

*Lepidium draba* L.

Lobularia

*Lobularia maritima*(L.) Desv.

Lunaria

*Lunaria annua* subsp. *pachyrhiza* (Borbás) Hayek

Raphanus

*Raphanus raphanistrum* L. subsp. *raphanistrum*

*Raphanus raphanistrum* subsp. *landra* (Moretti ex DC.) Bonnier & Layens

Rapistrum

*Rapistrum rugosum* (L.) All.

Rorippa

*Rorippa amphibia* (L.) Besser

Sisymbrium

*Sisymbrium officinale* (L.) Scop.

### Capparaceae

Capparis

*Capparis spinosa* L.

### Resedaceae

Reseda

*Reseda alba* L.

## Caryophyllales

### Amaranthaceae

Amaranthus

*Amaranthus deflexus* L.

*Amaranthus hypochondriacus* L.

*Amaranthus retroflexus* L.

Chenopodium

*Chenopodium album* L.

*Chenopodium suecicum* J. Murr

Chenopodiastrum

*Chenopodiastrum murale* (L.) S. Fuentes, Uotila & Borsch

Dysphania

*Dysphania ambrosioides* (L.) Mosyakin & Clemants

Lipandra

*Lipandra polysperma* (L.) S. Fuentes, Uotila & Borsch

### Basellaceae

Anredera

*Anredera cordifolia* (Ten.) Steenis

### Caryophyllaceae

Arenaria

*Arenaria leptoclados* (Rchb.) Guss.

Cerastium

*Cerastium brachypetalum* Desf. ex Pers.

*Cerastium glomeratum* Thuill.

*Cerastium pumilum* Curt.

Petrorhagia

*Petrorhagia prolifera* (L.) P. W. Ball & Heywood

Polycarpon

*Polycarpon tetraphyllum* (L.) L.

Sagina

*Sagina apetala* Ard.

Silene

*Silene baccifera* (L.) Roth

*Silene dioica* (L.) Clairv.

*Silene flos-cuculi* (L.) Greuter & Burdet

*Silene gallica* L

*Silene latifolia* subsp. *alba* (Miller) Greuter & Burdet

Stellaria

*Stellaria media* (L.) Vill.

*Stellaria neglecta* Weihe

### Phytolaccaceae

Phytolacca

*Phytolacca americana* L.

### Polygonaceae

Polygonum

*Polygonum aviculare* L.

Persicaria

*Persicaria maculosa* S. F. Gray subsp. *maculosa*

Fallopia

*Fallopia convolvulus* (L.) A. Löve

Rumex

*Rumex acetosa* L.

*Rumex conglomeratus* Murray

*Rumex pulcher* L.

*Rumex sanguineus* L.

## Cornales

### Cornaceae

Cornus

*Cornus sanguinea* L.

### Hydrangeaceae

Hydrangea

*Hydrangea arborescens* L.

*Hydrangea aspera* D. Don

*Hydrangea macrophylla* (Thunb.) Ser.

Philadelphus

*Philadelphus coronarius* L.

## Ericales

### Primulaceae

Cyclamen

*Cyclamen hederifolium* Aiton

*Cyclamen repandum* Sm.

Lysimachia

*Lysimachia arvensis* (L.) U. Manns & Anderb. subsp. *arvensis*

### Theaceae

Camellia

*Camellia japonica* L.

### Acanthaceae

Acanthus

*Acanthus mollis* L.

## Lamiales

### Bignoniaceae

Jacaranda

*Jacaranda mimosifolia* D. Don

### Lamiaceae

Ajuga

*Ajuga reptans* L.

Ballota

*Ballota nigra* subsp. *foetida* (Vis.) Hayek

Clinopodium

*Clinopodium nepeta* (L.) Kuntze

*Clinopodium nepeta subsp. spruneri*(Boiss.) Bartolucci & F.Conti

*Clinopodium vulgare* L. subsp. *vulgare*

Lamium

*Lamium flexuosum* Ten.

*Lamium purpureum* L.

Melissa

*Melissa officinalis* L. s.l.

Prunella

*Prunella vulgaris* L.

*Prunella laciniata* L.

Salvia

*Salvia officinalis* L.

*Salvia verbenaca* L.

*Salvia pratensis* L.

Stachys

*Stachys sylvatica* L.

Teucrium

*Teucrium scordium* subsp. *scordioides* (Schreb.) Arcang.

### Oleaceae

Fraxinus

*Fraxinus excelsior* L.

*Fraxinus ornus* L.

Jasminum

*Jasminum officinale* L.

Ligustrum

*Ligustrum japonicum* Thunb

*Ligustrum ovalifolium* Hassk.

*Ligustrum sinense* Lour.

Phillyrea

*Phillyrea latifolia* L.

### Orobancaceae

Orobanche

*Orobanche hederae* Duby

### Plantaginaceae

Antirrhinum

*Antirrhinum majus* L.

Antirrhinum

*Antirrhinum siculum* Mill.

*Antirrhinum tortuosum* Bosc ex Vent.

Cymbalaria

*Cymbalaria muralis* P. Gaertner, B. Mayer et Schreb.

Linaria

*Linaria purpurea* (L.) Miller

*Linaria vulgaris* Miller

Plantago

*Plantago lanceolata* L. subsp. *lanceolata*

*Plantago major* L.

Veronica

*Veronica arvensis* L.

*Veronica franciscana* (Eastw.) Howell, Raven & Rubtzoff

*Veronica persica* Poir.

*Veronica polita* Fries

### Scrophulariaceae

Scrophularia

*Scrophularia canina* subsp. *bicolor* (Sibth. & Sm.) W. Greuter

*Scrophularia nodosa* L.

*Scrophularia peregrina* L.

Verbascum

*Verbascum blattaria* L.

*Verbascum pulverulentum* Vill.

### Verbenaceae

Verbena

*Verbena officinalis* L.

## Solanales

### Convolvulaceae

Calystegia

*Calystegia silvatica* (Kit.) Griseb.

Convolvulus

*Convolvulus arvensis* L.

Cuscuta

*Cuscuta* sp.

### Solanaceae

Datura

*Datura stramonium* L.

Salpinchroa

*Salpinchroa origanifolia* (Lam.) Baillon

Solanum

*Solanum dulcamara* L.

*Solanum nigrum* L.

## Gentianales

### Apocynaceae

Araujia

*Araujia sericifera* Brot.

Nerium

*Nerium oleander* L.

Vinca

Vinca minor L.

*Vinca major* L.

Centaurium

*Centaurium erythraea* Rafn subsp. *erythraea*

### Rubiaceae

Galium

*Galium album* Mill.

*Galium aparine* L.

*Galium divaricatum* Pourr. ex Lam.

*Galium murale* (L.) All.

Rubia

*Rubia peregrina* L.

Sherardia

*Sherardia arvensis* L.

## Boraginales

### Boraginaceae

Anchusella

*Anchusella cretica* (Mill.) M. Bigazzi, E. Nardi & F. Selvi

Echium

*Echium italicum* L.

*Echium plantagineum* L.

*Echium vulgare* L.

Lycopsis

*Lycopsis arvensis* L. subsp. *arvensis*

Myosotis

*Myosotis arvensis* (L.) Hill

*Myosotis ramosissima* Rochel

Symphytum

*Symphytum bulbosum* C. Schimper

### Heliotropiaceae

Heliotropium

*Heliotropium europaeum* L.

## Aquifoliales

### Aquifoliaceae

Ilex

*Ilex latifolia* C. P. Thunb. ex A. Murray

## Asterales

### Asteraceae

Achillea

*Achillea nobilis* L.

Anacyclus

*Anacyclus clavatus* (Desf.) Pers.

Anthemis

*Anthemis arvensis* subsp. *sphacelata* (C. Presl) R. Fernandes

*Anthemis cotula* L.

Arctium

*Arctium minus* (Hill) Bernh.

Artemisia

*Artemisia annua* L.

*Artemisia campestris* subsp. *variabilis* (Ten.) Greuter

*Artemisia vulgaris* L.

Bellis

*Bellis annua* L.

*Bellis perennis* L.

Carduus

*Carduus pycnocephalus* L. subsp. *pycnocephalus*

Centaurea

*Centaurea jacea* L.

*Centaurea nigrescens* subsp. *neapolitana* (Boiss.) Dostál

Cichorium

*Cichorium intybus* L.

Cirsium

*Cirsium vulgare* (Savi) Ten.

Coleostephus

*Coleostephus myconis* (L.) Reichenb. Fil.

Crepis

*Crepis vesicaria* subsp. *taraxacifolia* (Thuill.) Thell.

Dittrichia

*Dittrichia viscosa* (L.) W. Greuter

Erigeron

*Erigeron bonariensis* L.

*Erigeron canadensis* L.

Eupatorium

*Eupatorium cannabinum* L. subsp. *cannabinum*

Galactites

*Galactites tomentosa* Moench

Galinsoga

*Galinsoga parviflora* Cav.

Gamochaeta

*Gamochaeta purpurea* (L.) Cabrera

Glebionis

*Glebionis segetum* (L.) Fourr.

Hypochaeris

*Hypochaeris radicans* L.

Lactuca

*Lactuca muralis* (L.) E. Mey.

*Lactuca serriola* L.

Lapsana

*Lapsana communis* L. subsp. *communis*

Matricaria

*Matricaria chamomilla* L.

Picris

*Picris hieracioides* L. s.l.

Pseudognaphalium

*Pseudognaphalium undulatum* (L.) O. M. Hilliard & B. L. Burtt

Reichardia

*Reichardia picroides* (L.) Roth

Roldana

*Roldana petasitis* (Sims) H.Rob. & Brettell

Senecio

*Senecio vulgaris* L.

Sonchus

*Sonchus asper* (L.) Hill

*Sonchus oleraceus* L.

*Sonchus tenerrimus* L.

Taraxacum

*Taraxacum officinale* Weber ex Wigg.

Xanthium

*Xanthium strumarium* subsp. *strumarium* (Moretti) Greuter

### Campanulaceae

Campanula

*Campanula erinus* L.

*Campanula trachelium* L.

Trachelium

*Trachelium caeruleum* L.

## Apiales

### Pittosporaceae

Pittosporum

*Pittosporum tobira* (Murray) Aiton fil.

### Apiaceae

Chaerophyllum

*Chaerophyllum temulum* L.

Conium

*Conium maculatum* L.

Daucus

*Daucus carota* L. subsp. *carota*

Ferula

*Ferula communis* L.

Foeniculum

*Foeniculum vulgare* Mill.

Sanicula

*Sanicula europaea* L.

Torilis

*Torilis arvensis* (Hudson) Link subsp. *arvensis*

*Torilis japonica* (Houtt.) DC.

Fatsia

*Fatsia japonica* (Thunb.) Decne. & Planch.

### Araliaceae

Hedera

*Hedera helix* L. s.l.

## Dipsacales

### Adoxaceae

Sambucus

*Sambucus nigra* L.

Viburnum

*Viburnum lantana* L.

*Viburnum opulus* L.

*Viburnum rhytidophyllum* Hemsl. ex Forb. & Hemsl.

*Viburnum tinus* L.

### Caprifoliaceae

Centranthus

*Centranthus ruber* (L.) DC. subsp. *ruber*

Lonicera

*Lonicera caprifolium* L.
